# Low-Power Ionically Tunable Bilayer MoS_2_ Synaptic Transistors

**DOI:** 10.1021/acs.nanolett.5c02372

**Published:** 2025-11-17

**Authors:** Or Levit, Emanuel Ber, Yair Keller, Boris Minkovich, Eilam Yalon

**Affiliations:** Viterbi Faculty of Electrical and Computer Engineering, 26747 Technion—Israel Institute of Technology, Haifa 32000, Israel

**Keywords:** electrochemical random-access memory, neuromorphic devices, non-volatile memory, two-dimensional semiconductors

## Abstract

Electrochemical random-access memory (ECRAM) devices offer the possibility of highly linear, energy-efficient conductance modulation via electrochemical doping. This makes them attractive as synaptic weights for neuromorphic applications. Herein, we demonstrate inorganic ECRAM transistors with two-dimensional (2D) bilayer MoS_2_ channels exhibiting both ionic and electrostatic gating. Our devices show electrochemical modulation of the channel conductance and electrostatic gating to achieve idle-state leakage currents of *I*_D_ < 100 fA (measurement limit). These unique capabilities are enabled by the 2D semiconductor channel, which supports both electrostatic (conventional field effect) and electrochemical, non-volatile gating. Unlike oxide-based ECRAMs, which typically operate in a degenerately doped regime, the crystalline 2D channel has a low baseline carrier density, which can be modulated electrostatically. Our devices demonstrate highly linear and symmetric training characteristics, evaluated using synaptic metrics commonly applied to memristive devices. These findings highlight the potential of 2D-based ECRAM devices for power-efficient synaptic electronics.

## Graphical abstract

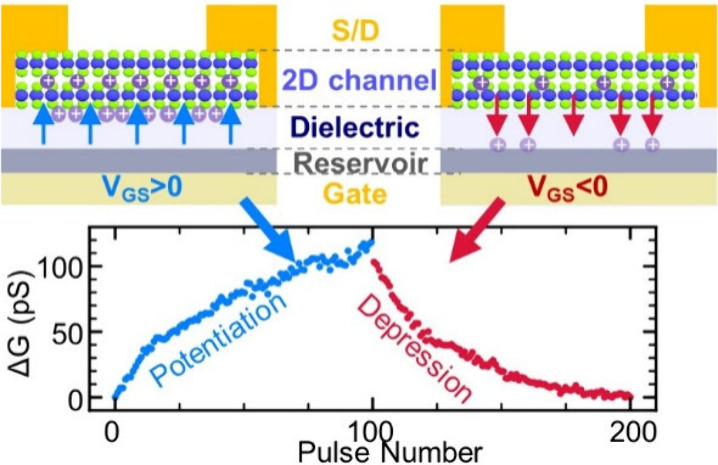

Electrochemical random-access memory (ECRAM) transistors are a class of emerging non-volatile memory (NVM) devices uniquely suited to store synaptic weights in neuromorphic hardware.^1,2^ They utilize the reversible transfer of ions between an ionic reservoir and the channel via field-induced migration through an insulating barrier. This motion is accompanied by a corresponding flow of electrons into the channel from the source and drain and effectively changes the channel doping.^3^ The direct control over channel doping allows ECRAMs to display conductance tunability with excellent linearity and symmetry.^4^ Previous studies have used ECRAMs as synaptic weight hardware to train artificial neural networks (ANNs) with high accuracy.^3,5^ Furthermore, ECRAMs are based on three-terminal architectures decoupling training (programming) and inference (read) paths, thus considerably improving energy efficiency,^6^ a crucial aspect in hardware-based neuromorphic computing.^7^

ECRAM transistors operate on the principle of electrochemical doping of the transistor channel through the insertion and extraction of mobile species. Lithium ions (Li^+^), oxygen vacancies, and protons are among the most commonly utilized species due to their relatively high mobility in solid-state electrolytes.^8–10^ ECRAMs also exhibit versatility in their potential channel materials, incorporating a range of materials, including oxides, organic semiconductors, various ionic conductors, and two-dimensional (2D) materials.^4,11–21^ Nonetheless, certain performance aspects of ECRAMs necessitate improvement, one of which is the programming speed. ECRAM programming involves mobilizing ions through the electrolyte when *V*_GS_ is applied, and redistribution of these ions inside the channel may occur once the gate bias is removed. These ionic effects are generally slower than other resistive switching technologies, which are based on nanoscale conductive filaments, and therefore limit the programming speed of ECRAMs.^22–25^ Moreover, many Li^+^-based ECRAM devices rely on ionic conductors, such as organic electrolytes and lithium phosphorus oxynitride (LiPON), which may not be fully compatible with standard CMOS conditions and may exhibit long-term stability issues.^3,26^

In a previous study, we proposed the employment of atomically thin semiconductors as ECRAM channel materials to enhance both programming times and energy efficiency in electrochemically activated devices by eliminating the ionic diffusion process within the channel.^27^ In this letter, we demonstrate ECRAM transistors based on bilayer MoS_2_ as a two-dimensional channel material, hence negating any thickness diffusion following the insertion of Li^+^ ions. MoS_2_ is one of most well-researched 2D semiconductors for next-generation, scaled, and back-end-of-line field-effect transistors (FETs).^28–30^ Moreover, the capacity of MoS_2_ films to intercalate Li^+^ ions in the interplanar gaps has been well-established,^31,32^ accompanied by an increase in conductance.^33,34^ These considerations make MoS_2_ the most suitable candidate to explore the possibility of ECRAMs with 2D channels. Bilayer MoS_2_ was chosen for this study to minimize the ionic redistribution associated with bulk channels while still providing the option to accommodate Li^+^ in the interplanar gap, which is not optional in monolayers. The devices that we used in this work demonstrate both ionic and electrostatic gating, enabling them to be used as non-volatile memory elements while acting as self-selecting elements.

We fabricated bottom-gated 2D ECRAMs similar to the scheme in Figure 1a–e by using the following steps. First, a combination of e-gun evaporation and wet etch was used to form Au bottom-gate structures. Then, radio-frequency (RF) magnetron sputtering was used to deposit a gate stack that consisted of 40 nm LiCoO_2_ (LCO) as an ionic reservoir and a ∼10 nm layer of AlO_*x*_ to serve as a diffusion medium and gate dielectric. For nanometric-scale devices, the reservoir can be also scaled in thickness. We therefore used a thin LCO layer that minimizes bulk diffusion effects while ensuring negligible changes to the Li^+^ concentration in the reservoir. After confirming that MoS_2_ is a bilayer n-type semiconductor (Figure S2 in the Supporting Information), we dry transferred it onto the chip and deposited Au source and drain electrodes via e-beam evaporation. Reactive ion etching (RIE) was used to remove excess channel material to set the channel dimensions of *W* = 3 μm and *L* = 1 μm, as displayed in the optical micrograph in Figure 1f. A more thorough description of the fabrication process is given in Figure S1 of the Supporting Information.

**Figure 1:**
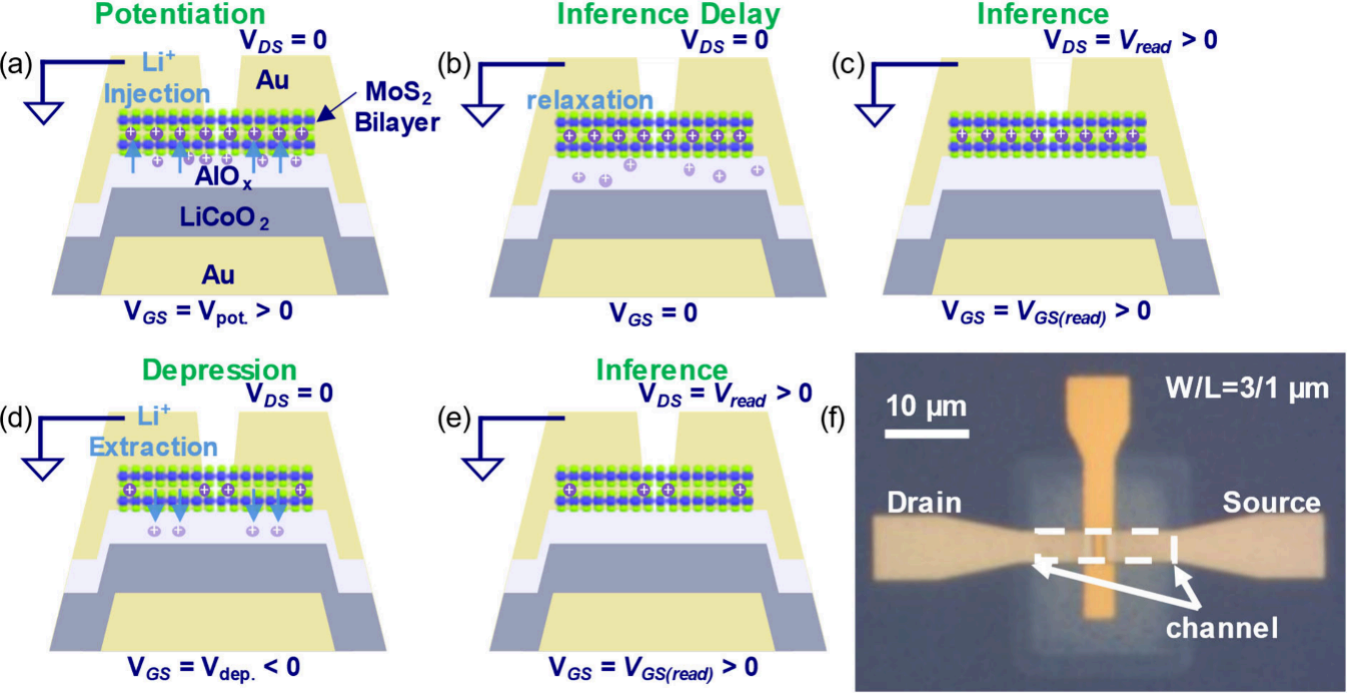
Schematic device structure and hypothesized operating mechanism of our 2D ECRAM: (a) Potentiation: Li^+^ ions are injected into the interplanar gap of the bilayer MoS_2_ using *V*_GS_ = *V*_pot_ > 0 pulses, increasing the channel carrier concentration and conductance. Positive *V*_GS_ also promotes surface accumulation of mobile cations at the AlO_*x*_/MoS_2_ interface. (b) Delay after potentiation: the idle state allows redistribution of the uninserted Li^+^ ions, the extent of which depends on the length of the delay period. (c) Inference: channel conductance is measured by application of *V*_DS_ pulses. *V*_GS_ is also applied to measure conductance while the transistor is on. (d) Depression: Li^+^ ions are extracted from the channel by application of *V*_GS_ = *V*_dep_ < 0 pulses, decreasing the channel carrier concentration and conductance, with no Li^+^ accumulation at the channel interface. (e) Inference after depression: a lower carrier concentration leads to lower channel conductance. (f) Top-view optical micrograph of a 3-terminal ECRAM device used in this work, before measurement.

We carried out electrical measurements under vacuum, using a Lake Shore Janis probe station and a Keysight B1500A semiconductor device analyzer equipped with high-resolution source/monitor units. First, we used DC *I*–*V* sweeps in high-resolution mode to obtain the transfer characteristics of the devices and determine the operational voltage window for programming/inference. Then, we performed pulsed measurements, applying a series of consecutive potentiation and depression *V*_GS_ pulses, as illustrated in Figure S3 in the Supporting Information. All programming pulses utilized a constant pulse width of 1 s, where positive potentiation gate bias, *V*_pot_, was used to increase the channel conductance, and negative depression bias, *V*_dep_, was used to decrease it. After a short delay time, *t*_delay_, in the range of 0.1–60 s, the state was read by measuring the channel conductance using a positive *V*_DS_ = V_read_ pulse. During the reading operation, *V*_GS_ was set to a positive value that kept the transistor in the on state. We then assess several key neuromorphic properties, including dynamic range, number of states, endurance, linearity, asymmetry, volatility, and power and energy consumption.

We extracted the transfer characteristics of the devices through *I*_D_–*V*_GS_ sweeps, as illustrated in Figure 2a. The results demonstrate electrostatic gating behavior with an on/off ratio of approximately 10^3^. At negative gate voltages, the measured drain current remains low (below ∼10 pA), increasing to above ∼100 pA around 2 V. This indicates that a positive gate bias is required to turn the channel on for conductance readout during inference. Such behavior could be advantageous in neuromorphic applications, enabling the device to function as a self-selective element in parallel programming schemes.^35^ We also observe a counterclockwise hysteresis loop, suggesting a mechanism different from the one observed in MoS_2_ before fabrication (Figure S2b in the Supporting Information). While we do not rule out the effect of trap sites in the AlO_*x*_/MoS_2_ interface, the changed hysteresis direction indicates an increase in carrier density in the channel during the reverse sweep. The origin of these electrons is most likely due to the accumulation of positive ions in the channel–dielectric interface through Li^+^ migration. To verify that it is also accompanied by electrochemical doping, we used a combination of *I*_D_–*V*_GS_ sweeps at varying sweep rates (Figure S4 in the Supporting Information) and consecutive identical sweeps (Figure S5 in the Supporting Information), where increased migration times and residual effects helped to confirm the occurrence of ionic doping.

**Figure 2:**
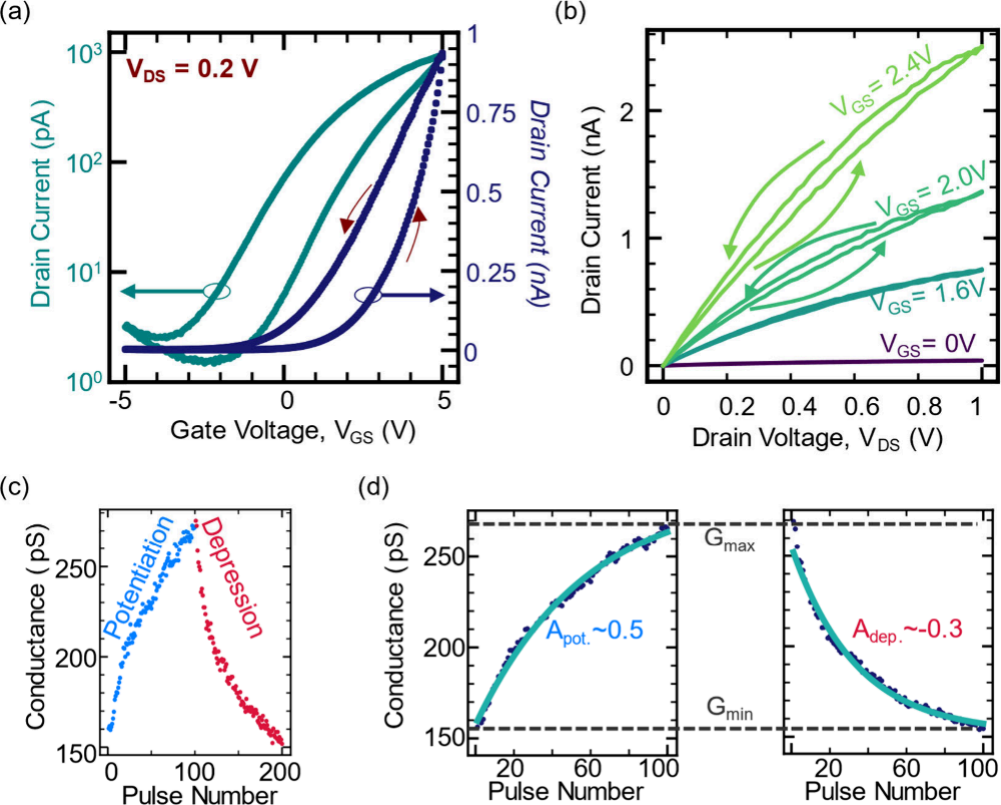
(a) Transfer curve *I*_D_–*V*_GS_ plot of a 2D ECRAM transistor prior to the application of training pulses, showing electrostatic gating (double sweep) on log (left) and linear (right) scales. The counterclockwise hysteresis suggests an increase in carrier density in the channel during measurement. (b) *I*_D_–*V*_DS_ measurements taken with varying *V*_GS_ values. At low gate bias, the transistor is off, resulting in no current throughout the measurement. Higher *V*_GS_ results in higher *I*_D_ and counterclockwise hysteresis. (c) Partial measurement of the channel conductance at *V*_*GS*_ = 2.5 V of a device following training cycles of 100/100 potentiation/depression steps of ±4.7 V, showing a dynamic range of ∼1.8. (d) Nonlinearity analysis of the mean-cycle behavior of the potentiation and depression phases. Channel width is W = 3 μm.

To further assess the channel behavior under operating conditions, we conducted a series of *I*_D_–*V*_DS_ measurements at varying gate biases (*V*_GS_ = 0, 1.6, 2.0, and 2.4 V), as shown in Figure 2b. Consistent with the transfer characteristics, negligible channel current is measured when the gate is unbiased, indicating that conduction is effectively suppressed in the absence of gate-induced field effects. At higher *V*_GS_ values, where the device operates in the “on” state, two key trends emerge. First, as expected, the channel current increases with increasing *V*_GS_, reflecting enhanced carrier density due to the field effect. The second trend is the evolution of a counterclockwise hysteresis loop as *V*_GS_ increases. The hysteresis direction indicates an increase in channel conductance, most likely the result of an increase in electron density in MoS_2_ due to vertical migration of positively charged Li^+^ ions toward the channel under positive gate bias, effectively doping it. This observation is consistent with the doping mechanism in Figure 2a and Figure S4.

For comparison, lateral Li^+^ migration would be expected to modulate doping primarily near the source, enhancing carrier injection. This effect should be more pronounced in the sub-threshold regime, producing counterclockwise hysteresis, whereas in the on state, where transport is dominated by drift current through the channel, a higher *V*_DS_ bias could instead deplete the Li^+^ concentration from the drain side, resulting in clockwise hysteresis. The output characteristics in Figure 2b, which exhibit counterclockwise hysteresis only at large *V*_GS_ and no hysteresis in the sub-threshold regime, therefore support predominantly vertical Li^+^ migration as the origin of the observed behavior.

For weight-update measurements, we applied a potentiation pulse with *V*_GS_ = 4.7 V to enable insertion of Li^+^ into the channel, followed by an inference (read) operation with *V*_GS_ = 2.5 V to electrostatically gate the channel while minimizing state drift. During inference, *V*_DS_ = 1.5 V was used to measure the channel conductance (*G*). For depression, the polarity of the programming pulse was reversed, while the inference pulse remained unchanged. A single programming cycle is shown in Figure 2c, consisting of a sequence of 100 potentiation and 100 depression pulses, using this scheme. All programming and inference pulses were 1 s wide. Based on previous work,^27^ we assume that shorter programming periods are possible, with the lower limit being the effective time constant for the ionic gate set by the ionic resistance and gate capacitance. The measurement reveals tens of discrete conductance states with a *G*_max_/*G*_min_ ratio of ∼1.8. Using similar measurements, we can apply previously reported NVM analysis methods^36^ to evaluate the programming nonlinearity and asymmetry. The conductance after each programming step, *G*_step_, is fitted according to *G*_step_ = *G*_0_ + *B*(1 – e^*P*/*A*^), where *G*_0_ is the initial conductance value of the training cycle, *P* is the relative step location in the programming cycle, and *A* and *B* are fitting parameters that relate to nonlinearity and the dynamic range, respectively. We fitted the nonlinearity model to the average per-step conductance values measured on the same device from Figure 2c and extracted the nonlinearity coefficients *A*_pot_ and *A*_dep_ for the potentiation and depression phases, respectively. As seen in Figure 2d, the fitting resulted in values of *A*_pot_ = 0.52 and *A*_dep_ = 0.28 and a programming asymmetry, defined as *S* = |*A*_pot_| – |*A*_dep_| = 0.24, making our devices comparable to leading neuromorphic devices.^1,36–38^ Figure S6 shows an expanded version of this measurement, including 6 similar training cycles, all with the same pulse parameters, showing consistently linear and symmetrical programming.

Figure 3 presents a 50 cycle subset of an endurance test, in which the ECRAM device was subjected to 1000 full training cycles, each consisting of 20 potentiation and 20 depression pulses at *V*_GS_ = ±4.7 V. In total, the device underwent 40 000 programming pulses without exhibiting any signs of failure. Given the relatively long duration of the programming pulses used in this study, the measurement represents an accumulated programming time exceeding 11 h, substantially longer than that reported in most comparable studies (see Table S1 in the Supporting Information). These results highlight the exceptional operational stability and durability of the device under sustained electrochemical stress.

**Figure 3:**
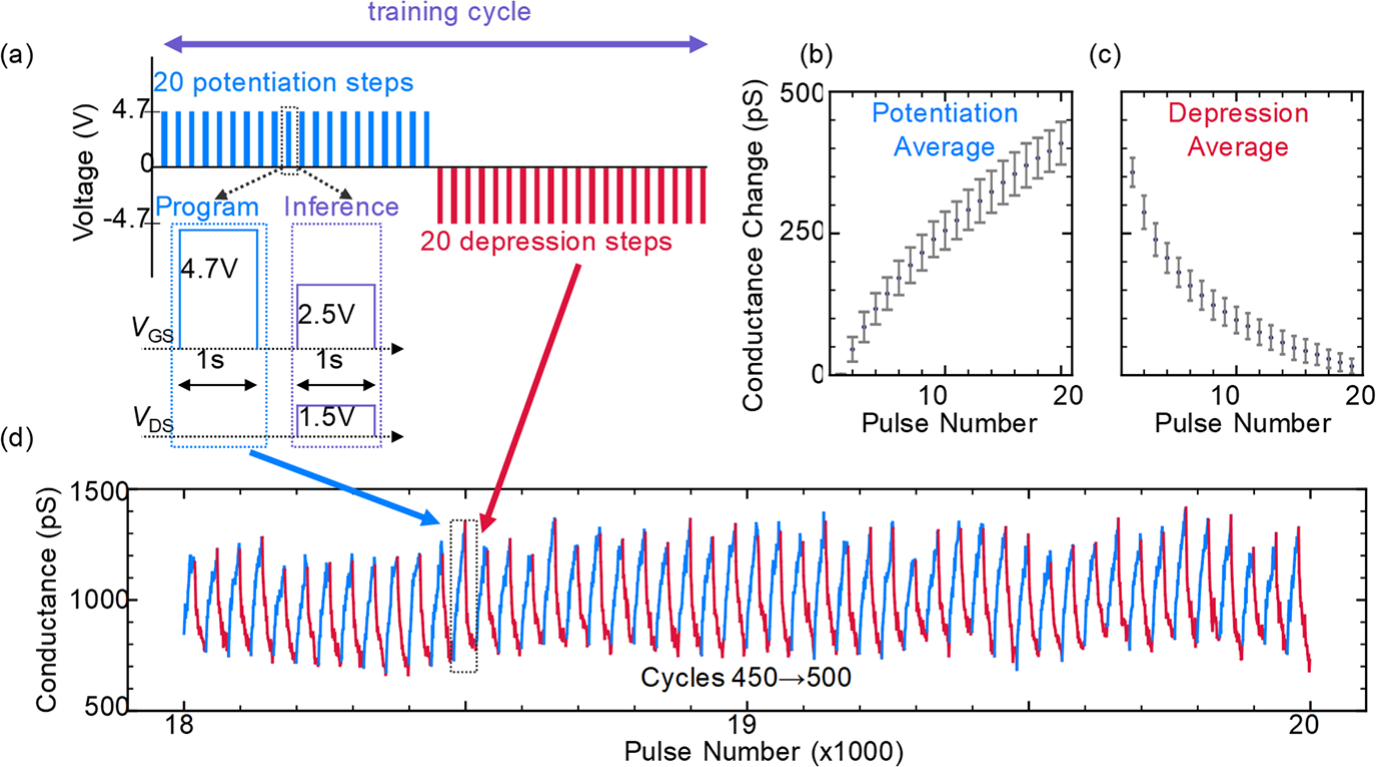
Cycling variability: channel conductance (*G*) vs number of pulses of an ECRAM device, which underwent a total of 40 000 1 s programming steps of ±4.7 V. (a) Voltage scheme for one of 1000 training cycles applied to the device. The same inference readout was used following a potentiation and depression pulse. Average conductance change from (b) potentiation and (c) depression phases along with their standard deviations and (d) subset of 50 measured cycles, displaying the cycle-to-cycle variation in the conductance.

We defined the response of the device as the absolute change in conductance from the minimum of each cycle and calculated the average response across all 1000 potentiation (Figure 3b) and depression (Figure 3c) phases. The resulting averages exhibit a highly linear response of ∼20 pS per programming pulse, which based on previous calculations correlates to an increase of ∼10^8^ cm^–2^ in the aerial Li^+^ concentration in the 2D channel via reversible ionic insertion and extraction.^27^ Nevertheless, the standard deviation across all measurements shows slight state overlap between consecutive programming steps. We assign this overlap to random measurement noise in high-resistance measurements, where the required current resolution between states is on the scale of 10^–12^ A, approaching the noise floor for pulsed measurements. The noise in measured current is evident in Figure 3d, which displays a segment of 50 out of the total 1000 applied training cycles. Despite this, the device response remains uniform across all 50 cycles shown, with a consistent dynamic range and overall behavior across the entire measurement window. More extensive cycling data and additional results from this measurement are provided in Figure S7 of the Supporting Information. The reported measurements are limited by test time, as the programming pulses are relatively long. The 2D AlO_*x*_ ECRAM is expected to exhibit high endurance, owing to the stability of its materials. Further improvements are anticipated through cell downscaling and reduced contact resistance, both of which lower the operating voltage and enable shorter pulse widths.

We evaluated the state retention using two complementary methods. In the first approach, a delay period, *t*_delay_, was introduced between the programming and inference pulses to allow for the relaxation of possible ionic double layers. The state is then determined by performing a single inference operation to complete a one-program/one-inference step, and the response after varying retention times is compared. The second method that we used to measure the retention involves multiple inference operations after each programming step using a one-program/multiple-inference scheme. Figure 4a–c shows one-program/one-inference results with relaxation times of 10, 60, and 300 s following either potentiation or depression. During these intervals, all device terminals were grounded (0 V). Due to time constraints, experiments involving longer delay periods employed fewer programming steps, and the programming voltage was 4 V. Across all conditions, the devices exhibited consistent programmability, with conductance changes on the order of ∼20 pS per pulse for both potentiation and depression. Considering that inference operations may affect the measured state by changing residual charges between inference operations or by further altering the conductance, we consider this first method to be a more reliable measure of intrinsic retention in this system since it employs a single inference pulse per state. In Figure 4d, we show five potentiation and five depression pulses of one-program/multiple-inference measurements, with inference operations spanning from seconds to 1000 s. Using this method, we can see clear state separation throughout the measurement, though some conductance drift is observed post potentiation.

**Figure 4:**
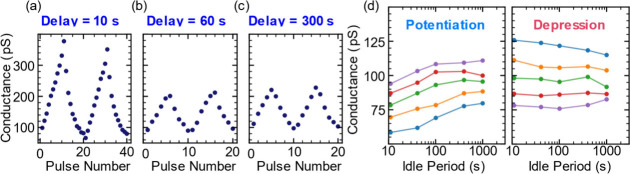
Non-volatility and retention: channel conductance of a device following potentiation/depression pulses of ±4 V with varying delay periods before inference. The method includes single-inference operation after (a) 10 s, (b) 30 s, and (c) 300 s and (d) sequence of consecutive inference operations at varying times up to 1000 s since programming. No significant data loss was recorded even after 1000 s. Note the different number of pulses between panel a and panels b and c. The conductance post potentiation shows an increase in the first two data points in time, possibly due to charge detrapping.

Finally, we evaluated the power consumption of our devices. With a channel area of 3 μm^2^, our devices exhibit power consumption in the order of 1 pW during programming steps and 100 pW per inference operation. These values are comparable to leading works in the field, as shown in Table S1 in the Supporting Information, where we benchmark some key efficiency metrics of bilayer MoS_2_ channel ECRAM devices to other state-of-the-art works. Specifically, the energy consumption can be reduced by the application of shorter programming pulses, as has been demonstrated in other ECRAMs.^4,8^ This time scaling is possible down to the lower boundary set by the effective RC delay of the device, which is on the order of ∼25 μs for this work. This limit can be reduced to the ∼nanosecond scale by decreasing device dimensions and improving the high contact resistance, which is estimated to be in the scale of ∼10^8^ Ω·μm in this device. A detailed discussion of the potential for shorter programming times and their impact on device performance is provided in section S9 of the Supporting Information. Inference power is primarily limited by the ability to probe conductance and can be further reduced when the readout is performed on-chip. The power efficiency of conductance modulation can be attributed to the atomically thin channel and the low baseline carrier concentration, which leads to a larger relative conductance increase with each programming step. Additionally, the relatively low overall conductance contributes to the reduced power consumption during the read operations. Furthermore, due to the ultralow idle-state gate leakage (∼10 fA), our ECRAMs are significantly less susceptible to state drift induced by the open-circuit potential (OCP). In conventional devices, OCP-related issues are often mitigated using external selector elements, which substantially increase circuit complexity and area overhead.^20,35,39^ This work, therefore, demonstrates promising characteristics for self-selective, power-efficient analogue synaptic applications.

In summary, this letter reports a CMOS-compatible, three terminal ECRAM transistor based on bilayer 2D MoS_2_. Importantly, our ECRAMs benefit from hybrid gating characteristics: a combination of electrostatic and electrochemical ionic gating. Electrostatic gating ensures low leakage currents and improved state retention, while electrochemical gating enables linear conductance modulation by the Li^+^ doping of the 2D channel. Specifically, we demonstrate that the devices achieve tens of discrete memory states with highly linear and symmetric programming, indicating strong potential for analog synaptic applications. These synaptic properties are sustained over 1000 programming cycles and 40 000 programming steps without failure and retained their programmed state for at least 1000 s. Based on our findings, ECRAMs featuring 2D material channels show strong promise for scaled, CMOS-compatible, low-power learning-in-memory applications, functioning as both synaptic devices and self-selective elements.

## Supplementary Material


