# Supplementary material for: Low-Power Ionically Tunable Bilayer MoS2 Synaptic Transistors
Source: Nano Lett. 2025 Nov 17;25(47):16580–5. doi: 10.1021/acs.nanolett.5c02372 (PMC12794164; doi:10.1021/acs.nanolett.5c02372)
Supplement: Supplementary file 1 [file nl5c02372_si_001.pdf]

Supporting Information

Low-Power Ionically Tunable Bilayer MoS_2 Synaptic Transistors

Or Levit, Emanuel Ber, Yair Keller, Boris Minkovich and Eilam Yalon*

Viterbi Faculty of Electrical and Computer Engineering, Technion – Israel Institute of Technology, Haifa, 32000, Israel (e-mail: eilamy@technion.ac.il).

* E-mail: eilamy@technion.ac.il

Table of Contents:

S1. Process and Device Schematics

S2: Structural and Electrical Characterization of Channel Material

S3: Measurement Setup for Pulsed Measurements

S4: Effect of the I_{DS}-V_{GS} Sweep Rate on Transfer Characteristics

S5. Consecutive Positive-Bias Sweeps

S6. Additional Programming Cycles – 100/100 Positive/Negative Steps

S7. Additional Endurance Cycles

S8: Power and Energy Comparison with Various ECRAM Mechanisms

S9: Programming Time Limitation

S1. Process and Device Schematics

Figure S1 shows the fabrication process flow for bilayer-MoS_2 ECRAM devices. First, a 100 nm SiO_2 layer was thermally grown on Si, followed by e-beam evaporation of 50 nm of Au to serve as the bottom-gate terminal. The transistor gates were defined via wet etch using a diluted version of commercial Au iodine-based etchant. Next, a combination of 40 nm $LiCoO_2$ (LCO) and 10 nm AlO_x were deposited using RF magnetron sputtering and lift-off. The nominal composition of the AlO_x target material was stoichiometric Al_2O_3, but due to the fact that only 10 nm were deposited on another oxide layer, the Al/O ratio may vary. The bilayer MoS_2 channel was dry-transferred from a uniform CVD-grown layer on SiO_2 and dry-etched using SF_6/O_2 plasma. Lastly, source and drain terminals of Ti/Au were added using e-beam evaporation and lift-off.

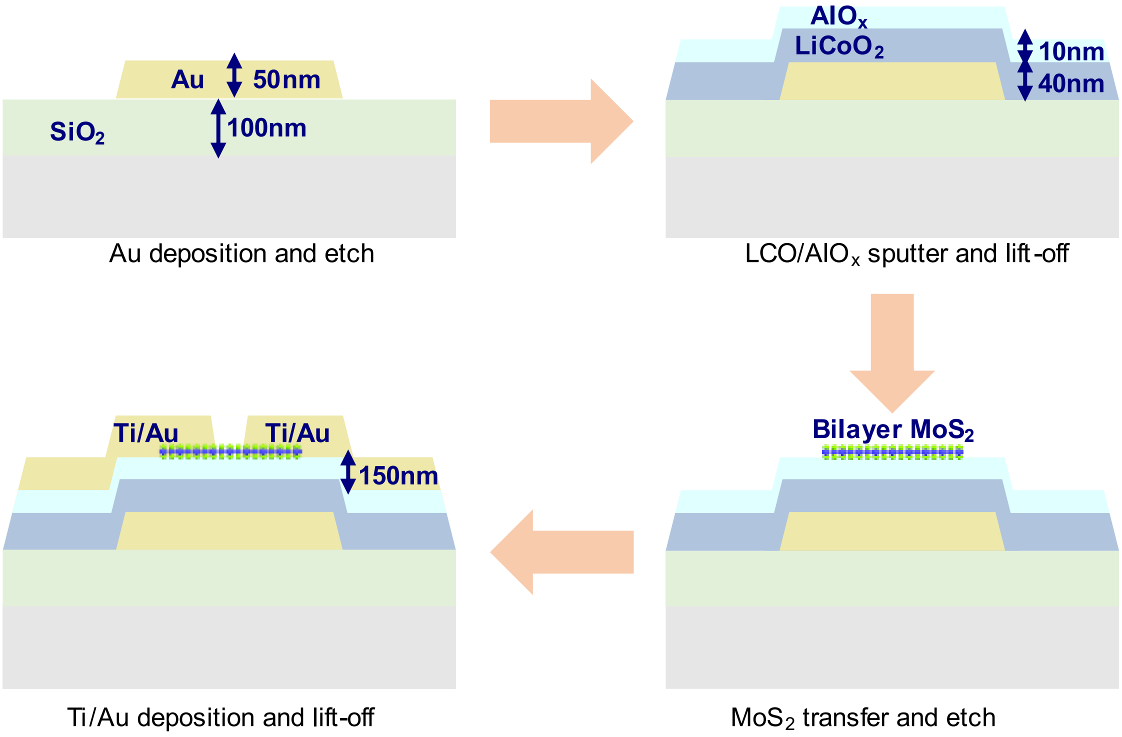

Figure S1: Fabrication scheme for the devices used in this work

S2: Structural and Electrical Characterization of Channel Material

Prior to device fabrication, we assessed the properties of the MoS_2 film using Raman spectroscopy and DC electrical measurements on the as-grown 2D material. Raman characterization was performed using a 532 nm excitation laser on a Si/SiO_2 (100 nm)/MoS_2 substrate. As shown in **Figure S2a**, the peak separation between the A_1g and E_2g modes is 22 (cm^{-1}), consistent with the presence of bilayer MoS_2, in agreement with previously reported values.[1] To evaluate the electrical behavior, we defined a channel and deposited Au contacts to form a back-gated field-effect transistor (FET), utilizing the 100 nm SiO_2 as the gate dielectric**. Figure S2b**, which shows I_D-V_{GS} measurement, clearly shows a field effect induced increase in channel current (notice the high gate voltage due to the thick dielectric oxide), confirming the behavior of a field effect transistor. The reverse sweep shows a slight reduction in measured current, resulting in clockwise hysteresis, which commonly associated with trapping of electrons during biasing[2,3].

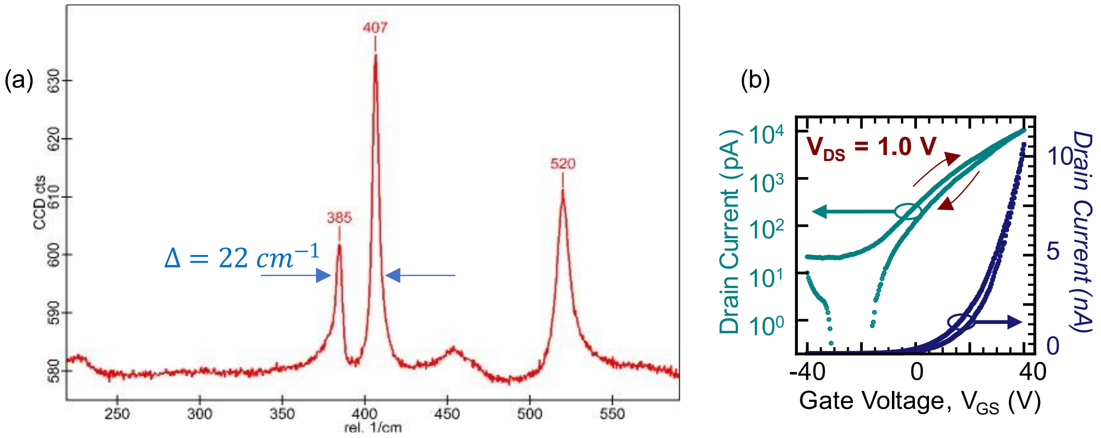

Figure S2. Preliminary characterization of bilayer MoS_2 film: (a) Raman spectroscopy, showing an A_{1g}-E_{2g} distance of 22 (cm^{-1}), confirming bilayer MoS_2. (b) I_D-V_{GS} sweep of $Si/SiO_2/MoS_2$ transistor, showing electrostatic gating of N-type transistor and a clockwise hysteresis loop.

S3. Pulsed Measurement Setup

Figure S3 displays the measurement setup for pulsed measurements in this work, along with the voltage-time waveform scheme. All pulsed measurements were done using a Keysight B1500A Semiconductor Device Analyzer equipped with a B1517A high-resolution source/monitor units (HRSMUs). Pulse width for both program and inference were 1 s, and the delay period was changed between measurements, as described in the main text. The source terminal was grounded during all measurements,

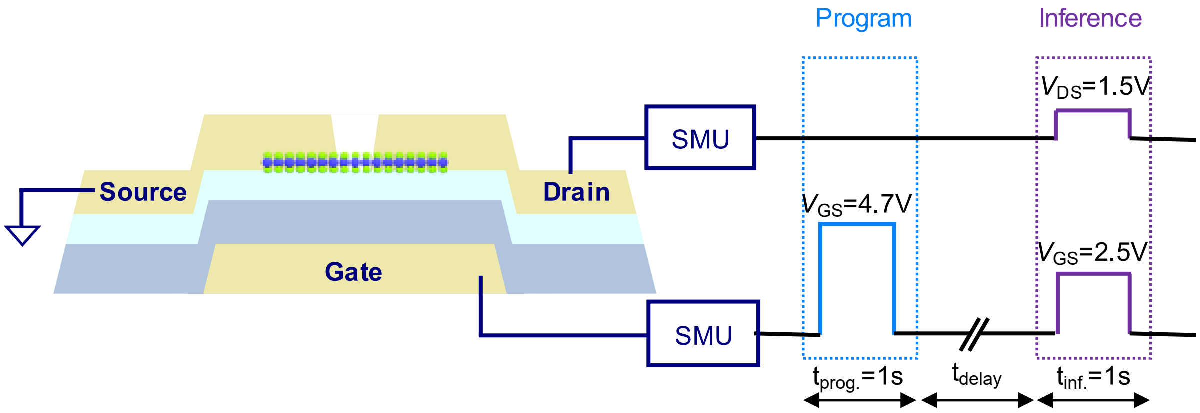

Figure S3. Voltage-time scheme for pulsed measurements of 2D ECRAMs, showing a single potentiation/inference step, with a generic delay period between them

S4: Effect of the I_{DS}-V_{GS} Sweep Rate on Transfer Characteristics

To investigate the origin of the counter-clockwise hysteresis observed in the I_{DS}-V_{GS} characteristics, we conducted a series of I_D-V_{GS} sweeps at varying rates. **Figure S4a–c** shows the results of a fast sweep (a), followed by a slower sweep (b), and then another fast sweep (c). All other measurement parameters, including voltage range, number of steps, and drain bias, were held constant across the three experiments, and between each sweep the device was given settling time of over 60 seconds. Two key observations can be made from these measurements. First, there is a progressive increase in channel current with each successive sweep, consistent with cumulative gate-induced doping of the channel. Second, the direction of the hysteresis loop evolves with sweep rate: at faster rates, a clockwise hysteresis is observed, whereas slower sweeps result in a counter-clockwise loop. The clockwise behaviour has been widely attributed in the literature to electron trapping in localized states within the oxide/MoS_2 interface[2,3], which suppress channel current during the reverse sweep. In contrast, the emergence of counter-clockwise hysteresis at slower sweep rates suggests a dynamic process that becomes more pronounced in longer timescales, most likely related to the field-effect driven migration of Li^+ ions towards the channel. This migration process can have either of two effects manifestations. First, cations can accumulate at the interface without electrochemically bonding to the MoS_2, creating a planar positive-charge concentration and effectively increase the driving force of electrons into the channel and lower the threshold voltage. This is a pure field effect, which should completely dissipate once V_{GS} is removed. The second way in which ionic migration affects the hysteresis is by doping the channel by electrochemical intercalation, increasing the electron concentration and the channel conductance in non-volatile fashion, as has previously shown elsewhere[4,5]. The residual increase in current even after allowing relaxation of field effects suggest that channel doping occurs during measurement.

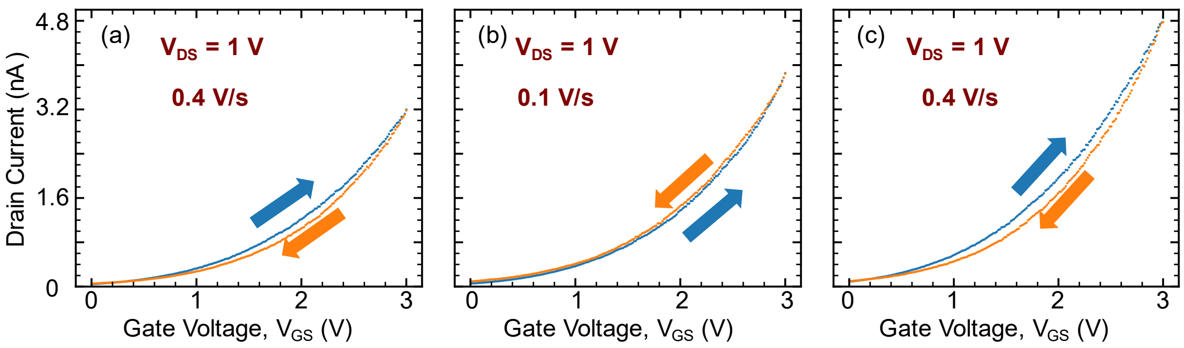

Figure S4. Three I_D-V_{GS} sweeps, at varying sweep rates. The faster sweeps (a, c) display a clockwise hysteresis loop, commonly associated with charge trapping in the gate oxide or at the interface. The slower sweep (b) exhibits a counter-clockwise hysteresis loop, indicating a more pronounced increase in channel conductance due to ionic effects.

S5. Consecutive Positive-Bias Sweeps

Figure S5 shows a series of consecutive I_D-V_{GS} sweeps. The sweeps included only positive V_{GS} values, thus enabling insertion of positive ions into the channel through the LCO/AlO_x stack, without promoting exertion in between sweeps. The channel current increases with each sweep, indicating an increase in carrier density due to ionic insertion, while the counter-clockwise hysteresis indicates the bias-induced increase in channel conductance. This measurement complements the one in Figure S4 in supporting the claim for electrochemical doping during DC measurements.

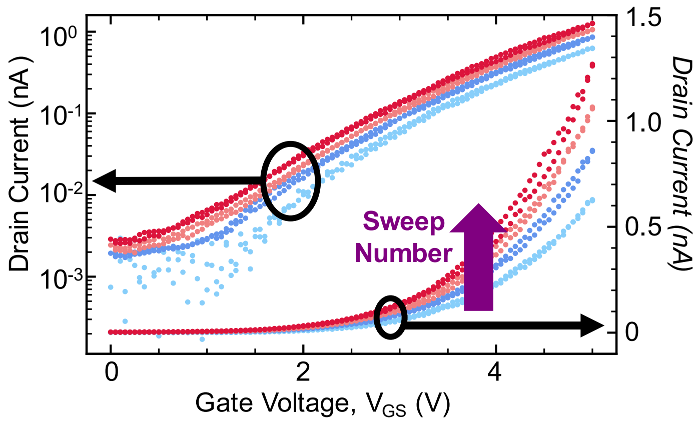

Figure S5. Consecutive I_D-V_{GS} sweeps from 0 V to 5 V, displaying counter-clockwise hysteresis and increased maximum current according to the sweep number. The obtained characteristics emphasize the increase in channel conductance due to positive ion doping, induced by positive gate voltage.

S6. Additional Programming Cycles – 100/100 Positive/Negative Steps

Figure S6 consists of a more comprehensive version of the results shown in **Figure 2c** in the main text. The main text includes a single programming cycle of 100/100 potentiation/depression steps, emphasizing the potential for linear and reversible conductance modulation. Here, a series of six programming cycles is included, exhibiting consistent programmability.

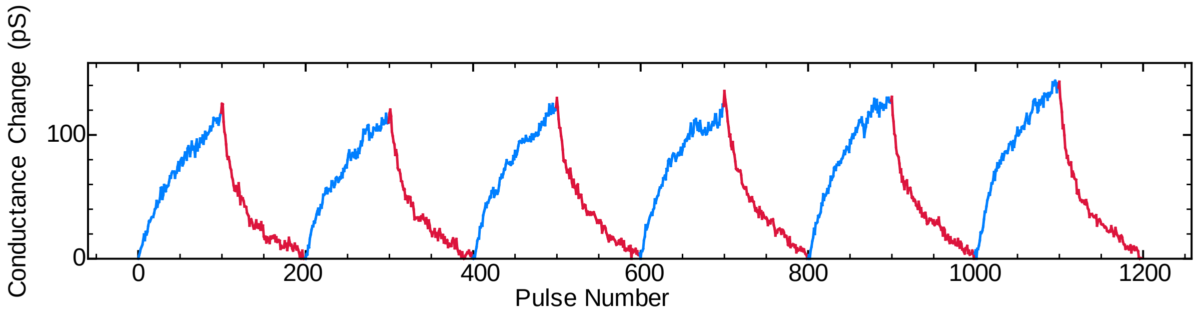

Figure S6. Conductance change of an ECRAM device throughout six training cycles of 100/100 potentiation/depression steps of ±4.7 V. Figure 2b in the main text presents an excerpt from this measurement.

S7. Additional Endurance Cycles

Figure S7 presents additional programming cycles from the same experimental dataset shown in **Figure 3d** of the main text. For clarity, the main text displays only 50 out of the 1000 programming cycles performed in this experiment. **Figure S7** includes 150 additional cycles, sampled from various points throughout the experiment. Although cycle-to-cycle variations are observed, the programming behavior remains consistent across the early, middle, and later cycles. Since each programming step involves applying $V_{GS} = \pm 4.7$ V, this measurement demonstrates a net endurance of over 5 hours under alternating high-amplitude voltage bias loading.

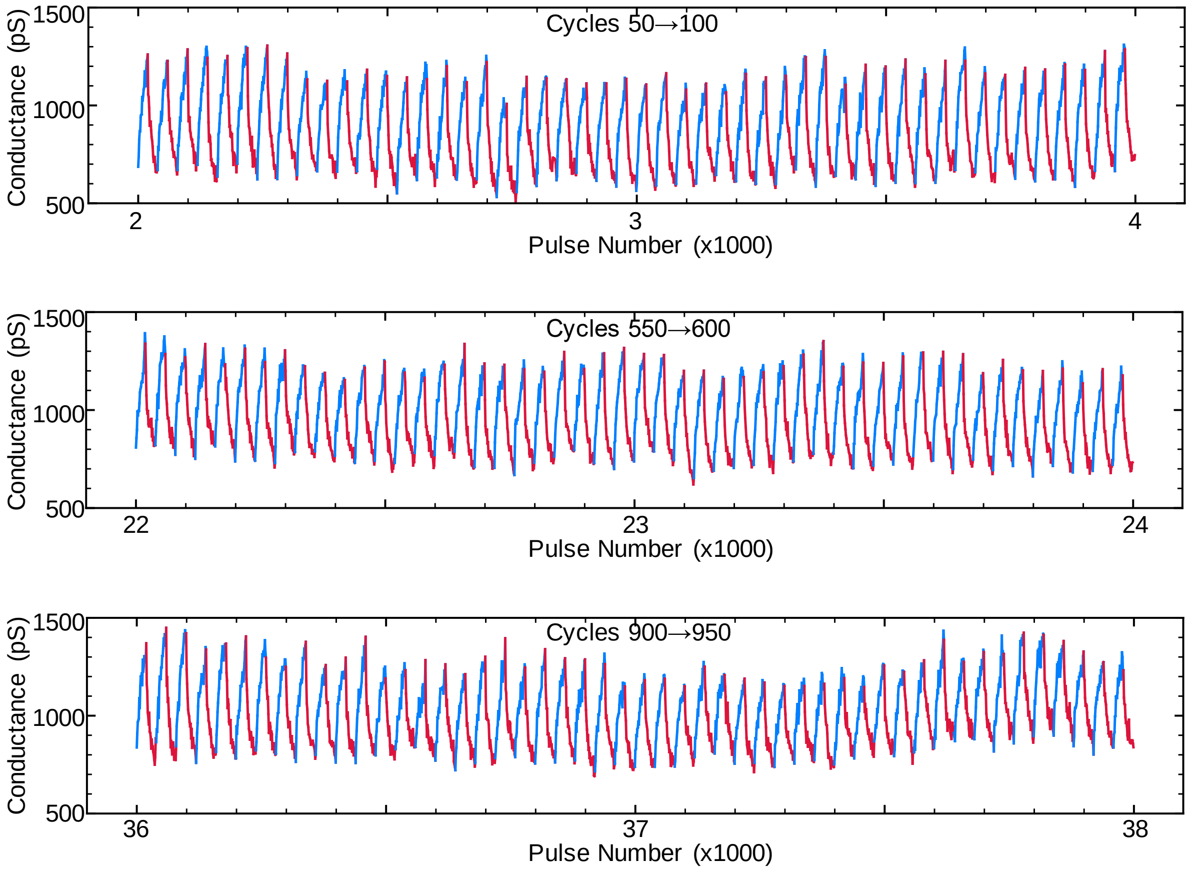

Figure S7. Three subsets, each consisting of 50 programming cycles including 20 potentiation and 20 depression steps, similar to Figure 3d in the main text. The selected subsets represent the start, middle, and end of this experiment.

S8: Power, Energy, and Retention Comparison with Various ECRAM Mechanisms

Table S1 compares the programming energy and average inference power of state-of-the-art ECRAM devices in literature with this work. We report average inference power, because it depends on the channel conductance which is modulated. In cases where only channel conductance was reported, we estimate power consumption by $P = GV^2$, and the reported inference data.

Regarding programming energy, it is important to recall that the lower bound of total programming energy is set by the capacitive charging and discharging of the device, namely $E \sim CV^2$. The energy consumption therefore strongly depends on device dimensions which determine its capacitance. Given the significant variation in device geometry, size and architecture (e.g., horizontal vs. vertical ECRAMs), it is necessary to normalize programming energy by channel area to enable fair comparisons.

In addition, state retention is included here as it directly affects the effective energy efficiency of memory devices. Devices with limited retention require frequent refresh operations, hence increasing the total power and energy consumption during device operation.

We report an inference power consumption in the scale of 10^2 pW/operation, among the lowest reported in ECRAM literature, as seen in **Table S1**. We attribute the power efficiency to the low conductance of the lightly doped MoS_2 channel, which can be gated both electrostatically (field effect) and by ionic doping. These mechanisms allow for modulation of the gate overdrive voltage, thereby efficiently controlling the channel current. However, our devices demonstrate relatively higher programming energy per operation, which we attribute to the use of comparatively long programming pulses in this work. Based on prior study[6], we hypothesize that programming energy can be further reduced by decreasing pulse duration significantly (orders of magnitude) which can be achieved by moderate increase in voltage, and by improving the device performance via contact engineering allowing more efficient charge injection and switching dynamics.

Table S1: comparison of specific programming energy, inference power consumption along with state retention times reported here to other ECRAM mechanisms

Device type	Mechanism	Diffusion Medium	Program Energy $\left(\frac{pJ}{state \cdot \mu m^2}\right)$	Inference Power $\left(\frac{pW}{operation}\right)$	Reported Retention (seconds)
This work	Li^+:MoS_2	AlO_x	1	10^2	10^3
G/LiPON/WO_3 [7]	Li^+:WO_3	LiPON	0.1	10^2	5
Pd:PSG:WO_3 [8]	H^+:WO_3	PSG	0.1	10^3	10^2
Si:LiPON:LCO [9]	Li^+:LCO	LiPON	0.1	10^6	Not reported
LFP:$LiClO_4$-PEO:C [10]	Li^+:graphene	PEO (horizontal)	<0.01	10^6	10^4

S9: Programming Time Limitation

All programming pulses in this work used a fixed duration of 1 s, which consequently affected the energy consumption. To assess the potential for scaling programming time (and by consequence, energy), we analyze the relevant limits and provide conservative estimates based on measured quantities.

The programming time is limited by the minimum time to electrostatically charge the gate:

$$\mathrm{t_{min}} \sim 5\mathrm{R_{series}C_{gate}} \,.$$

Since we lack direct measurements of these parameters, we use conservative approximations that likely overestimate both terms, yielding an upper bound on t_{min}.

We estimate C_{gate} as a parallel-plate capacitor with a relative dielectric constant of $\varepsilon_r \approx 10$:

$$C_{gate} = \frac{\epsilon_r \epsilon_0 L_{channel} W_{channel}}{t_{AlO_x}} \approx \frac{10 \cdot 8.85 \cdot 10^{-18} \left(\frac{F}{\mu m}\right) \cdot 3(\mu m^2)}{0.01\ (\mu m)} \approx\ 10^{-14}\ F$$

The series resistance includes both channel and contact contributions, because a sufficient amount of charge must reach the channel which acts as a counter electrode to the gate. To obtain a conservative upper bound, we use the total effective resistance ($\sim V_{DS}/I_D$) extracted from the forward sweep in the transfer curve in Figure 2a, at $V_{GS} = 4$ V:

$$R_{channel} = \frac{V_{DS}}{I_{D(@V_{GS=4V})}} = \frac{0.2\ V}{\sim 400\ pA} \approx\ 5 \cdot 10^8\ \Omega$$

These values yield:

$$t_{min} \sim\ 25\ \mu s$$

This limit is specific to our device and can be reduced significantly by improving contact resistance (R) and reducing device dimensions (C).

References

(1) Li, H.; Zhang, Q.; Yap, C. C. R.; Tay, B. K.; Edwin, T. H. T.; Olivier, A.; Baillargeat, D. From Bulk to Monolayer MoS_2: Evolution of Raman Scattering. *Adv. Funct. Mater.* **2012**, *22* (7), 1385–1390. https://doi.org/10.1002/adfm.201102111.

(2) Cho, A.-J.; Yang, S.; Park, K.; Namgung, S. D.; Kim, H.; Kwon, J.-Y. Multi-Layer MoS2 FET with Small Hysteresis by Using Atomic Layer Deposition Al2O3 as Gate Insulator. *ECS Solid State Lett.* **2014**, *3* (10), Q67–Q69. https://doi.org/10.1149/2.0111409ssl.

(3) Guo, Y.; Wei, X.; Shu, J.; Liu, B.; Yin, J.; Guan, C.; Han, Y.; Gao, S.; Chen, Q. Charge Trapping at the MoS2-SiO2 Interface and Its Effects on the Characteristics of MoS2 Metal-Oxide-Semiconductor Field Effect Transistors. *Appl. Phys. Lett.* **2015**, *106* (10). https://doi.org/10.1063/1.4914968.

(4) Xiong, F.; Wang, H.; Liu, X.; Sun, J.; Brongersma, M.; Pop, E.; Cui, Y. Li Intercalation in MoS2: In Situ Observation of Its Dynamics and Tuning Optical and Electrical Properties. *Nano Lett.* **2015**, *15* (10), 6777–6784. https://doi.org/10.1021/acs.nanolett.5b02619.

(5) Xiong, F.; Yalon, E.; McClellan, C. J.; Zhang, J.; Aslan, O. B.; Sood, A.; Sun, J.; Andolina, C. M.; Saidi, W. A.; Goodson, K. E.; Heinz, T. F.; Cui, Y.; Pop, E. Tuning Electrical and Interfacial Thermal Properties of Bilayer MoS2via Electrochemical Intercalation. *Nanotechnology* **2021**, *32* (26). https://doi.org/10.1088/1361-6528/abe78a.

(6) Levit, O.; Ber, E.; Dahan, M. M.; Keller, Y.; Yalon, E. Ionic-Electronic Dynamics in an Electrochemical Gate Stack toward High-Speed Artificial Synapses. *Appl. Phys. Lett.* **2023**, *123* (21). https://doi.org/10.1063/5.0169127.

(7) Tang, J.; Bishop, D.; Kim, S.; Copel, M.; Gokmen, T.; Todorov, T.; Shin, S.; Lee, K. T.; Solomon, P.; Chan, K.; Haensch, W.; Rozen, J. ECRAM as Scalable Synaptic Cell for High-Speed, Low-Power Neuromorphic Computing. *Tech. Dig. - Int. Electron Devices Meet. IEDM* **2019**, *2018-Decem*, 13.1.1-13.1.4. https://doi.org/10.1109/IEDM.2018.8614551.

(8) Onen, M.; Emond, N.; Wang, B.; Zhang, D.; Ross, F. M.; Li, J.; Yildiz, B.; Del Alamo, J. A. *Nanosecond Protonic Programmable Resistors for Analog Deep Learning*. https://www.science.org.

(9) Fuller, E. J.; Gabaly, F. E.; Léonard, F.; Agarwal, S.; Plimpton, S. J.; Jacobs-Gedrim, R. B.; James, C. D.; Marinella, M. J.; Talin, A. A. Li-Ion Synaptic Transistor for Low Power Analog Computing. *Adv. Mater.* **2017**, *29* (4), 1–8. https://doi.org/10.1002/adma.201604310.

(10) Sharbati, M. T.; Du, Y.; Torres, J.; Ardolino, N. D.; Yun, M.; Xiong, F. Low-Power, Electrochemically Tunable Graphene Synapses for Neuromorphic Computing. *Adv. Mater.* **2018**, *30* (36), 1–6. https://doi.org/10.1002/adma.201802353.
